# The Willingness of the Elderly to Choose Nursing Care: Evidence From in China

**DOI:** 10.3389/fpsyg.2022.865276

**Published:** 2022-03-11

**Authors:** Chengcheng Wang, Fanyu Zhang, Chao Pan, Shuyi Guo, Xianghong Gong, Dong Yang

**Affiliations:** ^1^School of Humanities and Foreign Languages, Qingdao University of Technology, Qingdao, China; ^2^School of Business Administration, Shandong University of Finance and Economics, Jinan, China; ^3^Department of Nursing, Nanyang Vocational College of Science and Technology, Nanyang, China; ^4^School of Statistics, Southwestern University of Finance and Economics, Chengdu, China

**Keywords:** nursing care, willingness, psychological and social support, intergenerational support, China

## Abstract

With the accelerating aging of the population and the worsening psychological conditions of older people, the traditional mode of family support for the elderly in China does not always meet the physical and psychological needs of the elderly, and more social support modes for the elderly are needed. Based on 3,513 valid questionnaires on the long-term care and protection needs of Chinese residents, this paper uses a logit regression model to analyze the factors influencing the willingness of the elderly to choose nursing care. The results show that intergenerational family support for the elderly is a significant psychological driver on the willingness of the elderly to choose nursing care. Compared with the elderly living with family, empty nesters or older people living alone are more inclined to select nursing care when they have difficulties taking care of themselves. The physical health of the elderly affects their willingness to choose nursing care, and elderly individuals with more hospitalizations are less likely to select nursing care. In addition, elderly females who are relatively young, have a high level of education, have a high income, have a nursing home near the residence, and are already covered by medical insurance are more willing to choose nursing care. The results of this study are of great importance for improving the medical services and aging care services for the elderly and providing theoretical support for alleviating the psychological and social pressure brought by population aging.

## Introduction

Currently, population aging has become a globally prominent problem. The *World Population Prospects* released by the United Nations (UN) in 2019 reports that the growth of the elderly population aged 65 years and older is the fastest. At present, this population accounts for approximately 9% of the global population, and it is expected to increase to 20% by 2050 (United Nations Department for Economic and Social Affairs, 2019). In the aging process, disabilities, and chronic diseases are closely related to the physical and psychological health of the elderly. The elderly population is significantly increasing, and the population aged 80 years and above will increase from 143 million to 426 million over the next 30 years. Epidemiological evidence suggests that stroke and poststroke cognitive impairment (PSCI) may significantly impact the needs for nursing care (Donnelly et al., 2020). The elderly are at a higher risk for multiple comorbidities, functional dependence, and cognitive impairment (Amblàs-Novellas et al., 2020). The physical and psychological health problems of the elderly brought by population aging increase the burden of family care.

In the process of modernization, the environment and health are closely related; therefore, environmental issues also affect the health of the elderly. Data reported in *STATE OF GLOBAL AIR/2018* indicate that more than 95% of the world’s population is breathing dirty air that exceeds the allowed particulate matter (PM) concentration in global air quality guidelines; notably, the situation is even more severe in underprivileged countries (Tian et al., 2020, 2022; Yang et al., 2020; Wan et al., 2021). In 2016, approximately 6.1 million deaths worldwide were attributable to air pollution, including stroke, heart disease, lung cancer, and chronic lung diseases (IHME [The Institute for Health Metrics and Evaluation], 2018). The Institute for Health Metrics and Evaluation (IHEI) proposes that air pollution has caused a substantial number of casualties worldwide and has caused many older people to seek care at hospitals. Reductions in living spaces for older people and the deterioration in air quality have affected the personal networks of the elderly (Gottlieb, 1985). Environmental issues have increased the incidence and types of chronic diseases, leading to a substantial increase in health expenditures (Moon and Choi, 2018) and creating new challenges for nursing care and public health systems.

The physical and psychological problems of the elderly brought by population aging and air pollution have increased the urgency and diversity of needs for elderly care, particularly in developing countries. According to *World Population Aging 2017*, by 2050, 79% of the population aged 60 years or above will live in developing countries and regions (UN [United Nations], 2017). The process of industrialization and urbanization in developing countries has accelerated the pace and scale of population aging, but the service level of elderly care has not met the growing demand for elderly care. Furthermore, the industrial structure of developing countries, where industry and manufacturing are dominant, creates more serious environmental problems. As a developing country with the largest elderly population, China entered an aging society in 1999. In the subsequent 20 years, population aging has shown a trend of rapid development. According to the National Bureau of Statistics of China, at the end of 2019, the population aged 60 years and older was 253.88 million, accounting for 18.1% of the total population, and there were 176.03 million people aged 65 years and older, accounting for 12.6% of the total population. According to relevant estimates, 2053 could be the peak for China’s aging population. At that time, the elderly population in China will reach 487 million, accounting for a quarter of the total elderly people in the world.

The typical family structure of “4 elderly, 1 couple, and 1 child,” the weakening of the traditional family support mode for the elderly, the changes in the social division of labor, and the reduction in the labor force population have all led to a substantial gap in elderly care. The World Health Organization (WHO) predicts that by 2050, 110.5 million people will need daily care, accounting for 6% of the total population. Of these, 66 million are older people aged 60 years and over, accounting for 59.7% of the people in need of daily care (WHO [World Health Organization], 2015a). The aging of China’s population is accompanied by the aging of the aged, chronic diseases, disability, and empty nesters. First, the percentage of very older people in the elderly population in China is gradually increasing. According to the national census, China’s elderly people aged 80 and above increased from 11.99 million to 35.8 million, The proportion of the elderly population aged 80 and above increases from 1.0 to 2.5%, an increase of 1.5 percentage points, which is significantly faster than the overall level worldwide (United Nations Department for Economic and Social Affairs, 2019). Older peopleSecond, there is little optimism regarding the health of Chinese older people. More than 180 million older people suffer from chronic diseases. As high as 75% of older people have one or more chronic diseases, and 50% have more than two chronic diseases. Chronic diseases account for 91.2% of elderly deaths in China and have a significant impact on health. Third, the increase in the number of elderly with disabilities and dementia increases the need for care. According to *The fourth sample survey on living conditions of urban and rural elderly in China* (2016), there were 40.63 million disabled and semi-disabled elderly in China, accounting for 18.3% of all older adults aged 60 years or older; this population will exceed 73 million in 2034. Fourth, unlike other countries, with modernization and urbanization, the number of empty nesters in China has increased year by year. In 2016, 50% of the Chinese elderly population was empty nesters, with over 70% in large and medium cities, creating challenges for traditional family support for the elderly. The care needs gradually diversify with aging. To meet the needs of the elderly in China regarding long-term care and medical care, different social support modes for elderly care are required.

Healthy aging is an essential indicator of the development and progress of human society. In 2015, the WHO released the *World report on aging and health*, which defines healthy aging as “the process of developing and maintaining the functional ability that enables well-being in older age”; the report emphasizes that “healthy aging is more than just the absence of disease. For most older people, the maintenance of functional ability has the highest importance” (WHO [World Health Organization], 2015b); furthermore, it suggests that long-term care is required when the elderly with severe disabilities cannot maintain their normal daily life without assistance. Long-term care is divided into nursing professional care and family nonprofessional care; the specialized long-term care provided by nursing homes is critical. As pointed out in the report, today’s society has realized that excessive reliance on family care may be detrimental to the well-being of the elderly; additionally, the burden to women, as traditional caregivers, is notable. Therefore, “debates will be needed about future reliance on families, the state or private sectors in caring for older persons.” At present, China is constructing an elderly care system based on family care, supported by community care, supplemented by nursing care, and combined with medical care. With the promulgation of relevant policies, nursing elderly care has gradually been accepted by people, and the number and scale of nursing homes have increased rapidly. Statistical Communique of the People’s Republic of China on the 2020 National Economic and Social Development shows that by the end of 2020, there were 38,000 nursing homes for the elderly, with 8,238,000 beds in nursing homes. However, behind the booming development of nursing homes, there is a gap in demand for elderly care and a high vacancy rate.

Therefore, it is of great significance to explore the factors influencing the willingness of the elderly to choose nursing care, to provide the necessary amount of nursing elderly care, to maintain the function ability of the elderly, and to improve the quality of life of the elderly. Research on factors influencing the willingness of the elderly in China to choose nursing care has mainly been addressed considering three dimensions: individual, family, and society. Zhang and Wei (2014) employed the data from “The survey on demands of the elderly for aging support services in Xicheng District” of Beijing, and adopted the Anderson Model as the analytical framework. The regression results indicated that those four kinds of factors are significantly associated with the intention to nursing care of the elderly without activities of daily living disability (ADL) disability. However, for the elderly with ADL disability, their intentions are highly related to tendency and enabling factors and demands for services (Zhang and Wei, 2014). Yu and Liao (2015) applied the ordered logistic model to take an empirical analysis on social old-age insurance treatment differences and willingness of institutions for old-age care from urban and rural residents, based on sample survey from urban and rural areas of South China, Central China, East China, North China and northeast area. The result indicates that the differences of social old-age insurance treatment affect the consumption decisions of individuals or families and their willingness of institution for old-age care. Luo et al. (2018) collected data from 641 elders aged 60 years old and above in six community health centers in Shanghai. They found that shame and adaptability of elders, and the services provided by nursing homes were the predictors of the elders’ willingness. Studies have shown that factors such as the elderly themselves, their families and community services have significant impacts on the willingness of the elderly to choose nursing care. However, in the previous studies, the small sample size or the choice of sampling method made the overall sample less representative and the sampling error larger.

In this paper, based on an analysis and discussion of the data generated by probability-proportional-to-size (PPS) sampling from 7 cities (Beijing, Guangzhou, Ningbo, Qingdao, Changchun, Chongqing, and Lanzhou) in China, the significant factors that influence the willingness of the elderly in China to choose nursing care were obtained. Based on the theory of healthy aging, relevant policies and recommendations are proposed according to the analysis results.

## Materials and Methods

### Data

In 2016, researchers for “Research on the Needs of Long-term Care Security in China” used the PPS sampling method to select 3,528 resident families from 24 communities, 6 blocks, and 3 districts in each of 7 cities in China, in order to comprehensively and systematically understand the living conditions of elderly people and their families as well as their needs and willingness to choose long-term care. The questionnaire was designed according to the following three steps: The first step was to gather information regarding the primary conditions of the respondents and their families. The second step was to collect information regarding the respondents’ health status and their needs and willingness to choose long-term care. The third step was to gather information regarding the respondents’ needs and willingness for long-term care financing. Respondent refers to the elderly individual in the selected family who answered the survey questions. Because some older people were unable to answer the questions themselves or needed their relatives to help provide their responses, spouses, children, or other relatives and friends who were familiar with the family situation were allowed to answer for the respondent.

The questionnaire contains seven parts. Part A obtained the basic information of the respondent. Part B obtained the family status of the respondent. Part C assessed the burden of medical expenses and medical insurance coverage of the respondent and his/her family. Part D obtained the death status of elderly relatives and friends. Part E obtained the health status of the respondent. Part F assessed the respondent’s needs and willingness for long-term care services. Part G assessed the respondent’s willingness to participate in long-term care insurance.

The method used by the research team to select the sample for the study was as follows: first, stratified sampling was used to select communities, blocks, and districts, and then systematic sampling was used to select households. Sampling was divided into five steps. In the first step, the 20 pilot provinces and cities in China implementing the long-term care insurance system were divided into 6 regions, i.e., North China, South China, East China, Northeast China, Southwest China, and Northwest China. Among them, 7 cities were selected as the sample cities. In the second and third steps, 24 communities in 6 blocks and 3 districts were selected from each city using the PPS sampling method. The fourth step was to determine the households with older people aged 50 years or older from household registration data to form the sampling frame. Then, 21 households were sampled from each community using systematic sampling. The research team stipulated that no less than 500 respondents should be in each city. In the fifth step, in each selected family, the research team interviewed an older person aged 50 years or older and a young person and middle-aged person present at the time of the survey. A total of 5,998 valid questionnaires were obtained, i.e., 3,513 questionnaires from the elderly and 2,388 questionnaires from young and middle-aged people. This study used data from 3,513 questionnaires obtained from elderly individuals to analyze and explore factors affecting the willingness of the elderly in China to choose nursing homes when they begin to have difficulties in taking care of themselves.

### Statistical Analyses

Among the elderly who provided the 3,513 valid questionnaires, 34.07% were male, and 65.93% were female; therefore, the male to female ratio was unbalanced. This imbalance may be that women’s average life expectancy is higher than that of men (Austad, 2006), which also confirms the trend of more older women than older men in China’s sixth census. Generally speaking, the status of women in the family or society is lower than that of men, and whether in the economic level or in the spiritual level, women and men both have different pension needs. When taking care of oneself begins to become difficult, approximately 50% of the elderly are willing to choose nursing homes, only approximately 30% choose care at home, and approximately 20% have not thought about receiving care at a nursing home; these data indicate that most older people in China have gradually accepted nursing care. More than 90% of older adults do not live with their children, indicating that the phenomenon of empty nesters in China is predominant among the elderly. Empty nesters are more likely to feel lonely and are at increased risk for depression (Cheng et al., 2015). Although the age of the respondents primarily ranged from 60 to 69 years old, the percentage of older people aged 80 years or older was also high, approximately 12.64%. Significantly older people are at an increased risk of having an unhealthy physical condition, increasing their burden of care (Bloom et al., 2015). Based on the survey, 35.55% of older people reported that a nursing home was located near their place of residence, 36.38% of older adults reported that no nursing home was nearby, and 28.07% of the elderly reported that they did not know if a nursing home was located nearby. To some extent, these data reflect some issues regarding nursing homes in China, such as a lack of a unified plan and layout, the feeling by community residents of a separation from life, and inadequate publicity. Combined with the proportion of the willingness to provide for the aged in institutions, some older people in China are influenced by traditional concepts and lack of planning for the future pension mode. The elderly without pension institutions near their residence will not take the initiative to understand the situation of pension institutions. From the perspective of insurance coverage for the elderly, only 3.3% of the elderly were not covered through social medical insurance, indicating that the elderly in China have a high medical insurance coverage rate. As the proportion of the elderly population in China has continued to increase, the government has paid more attention to and reformed the medical insurance system for older people, and the enthusiasm of older people regarding their participation in the insurance system has dramatically increased (Table 1).

**TABLE 1:** Questionnaire sample information.

Variable	Type	Category	Count	Percent
Nursing Care Willingness	Categorical	Willingness	1,842	52.43
		No Willingness	999	28.44
		Never thought	672	19.13
Gender	Categorical	Male	1,197	34.07
		Female	2,316	65.93
Age(years)	Continuous	<60	746	21.24
		60-69	1,437	40.90
		70-79	886	25.22
		≥80	444	12.64
Marital Status	Categorical	Married	2,655	75.58
		Widowed	721	20.52
		Divorced	94	2.68
		Never married	43	1.22
Education	Categorical	<Primary	314	8.94
		Primary	769	21.89
		Year 6 to 12	2,006	57.10
		>Year 12	424	12.07
Monthly Income	Continuous	<1,000	1,014	28.86
		1,000-2,999	1,479	42.10
		≥3,000	1,020	29.04
Coresidence	Categorical	Coresidence	274	8.13
		Non-Coresidence	3,098	91.87
Hospital Times	Categorical	0	2,508	71.39
		1	676	19.24
		2	213	6.06
		3	66	1.88
		4	50	1.42
Nursing Care Nearby	Categorical	Yes	1,249	35.55
		No	1,278	36.38
		Unknown	986	28.07
Insurance	Categorical	Insured	3,397	96.70
		Uninsured	116	3.30

**FIGURE 1:**
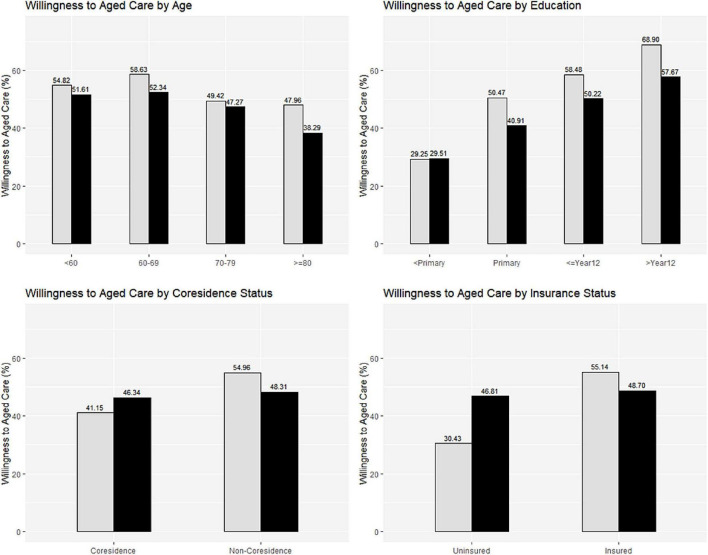
Willingness to aged care by demography.

To more clearly and intuitively demonstrate the changes in the willingness of the elderly in China to choose nursing care, this paper visualized the data. In Figure 1, gray represents females, and black represents males. It can be seen from the figure that with increasing age, the willingness of the elderly aged 60 and above in China to choose nursing homes gradually decreases. Meanwhile, the elderly aged 50 to 59 are less willing to choose nursing homes than those aged 60 to 69. In particular, 54.4% of women chose nursing homes, which is 6% higher than the percentage of men who chose nursing homes, indicating that there is a gender difference in the willingness of the elderly to choose nursing care. Compared with women, as they increase in age, men were less willing to go to a nursing home. After grouping the elderly based on gender and education level, there were also significant differences among different groups regarding the willingness of the elderly to choose nursing care. With the increase in years of education, the willingness of the elderly to choose nursing care increased significantly, and the increase in the willingness of elderly females was notable. Notably, there was no gender difference in the willingness of uneducated older people to choose nursing care. However, among educated older adults, the proportion of women who chose nursing care was much higher than that of men, approximately 10% higher than that of men. Generally, women play a long-term role as caregivers for family members. Therefore, when these women age, experience reduced physical health, and cannot take care of their families, they often think that they cannot receive good care from their families; therefore, they are more inclined to choose social care (Wang and Liu, 2020). Additionally, the more educated the elderly are, the more tolerant and open they are, and the more likely they will accept untraditional modes of social support for care. Under normal circumstances, the higher one’s education level, the higher is that individual’s economic income. For uneducated older women, their financial income may not be sufficient to cover the expenses of a nursing home; therefore, their willingness to choose nursing elderly care is not much different from that of their male counterparts. However, for older women with a high level of education, covering the expenses of a nursing home is not an obstacle regarding the choice to receive care at a nursing home, thus creating a difference between men and women. In addition, compared with men, women’s financial literacy and financial security are relatively low (Xue et al., 2019, 2020, 2021). When they have more income, they are more likely to spend for themselves rather than make other investments.

Whether parents and children live together has a certain impact on the elderly’s pension willingness. Overall, the elderly who do not live with their children are more inclined to provide for the aged in institutions. This may be due to the function of family support in the family where parents and children live together is stronger, and the elderly’s demand for socialized pension is not high. For most of the empty female nesters, without children at their side makes their mother’s role gradually weakened, and they are more likely to have a sense of loneliness. Therefore, whether or not to live with their children will have a more significant impact on the psychology and life of elderly women. The older women are more inclined to provide for the aged in institutions when they haven’t childless company. At the same time, the elderly who participate in social medical insurance are more willing to choose nursing pension, especially female elderly. 43% and 55. 14% of the older women who did not participate in the insurance and participated, respectively, which was about 25% different. Interestingly, whether parents live with their children or whether they are insured does not have a significant impact on the male elderly, but it is pronounced for the female elderly. This may be because, compared with men, older women are more vulnerable to insecurity, more economically dependent, and less likely to have access to long-term care resources.

### Regression Modeling

To examine the effect of each factor on the willingness of the elderly to choose nursing care, this paper adopted the following logit regression model, in which the subscript *i* represents individual *i*.


$$\begin{array}{c} \text{PR}\left(Nur_{i}=1|Cor_{i},Hos_{i},\ldots ,Nea_{i}\right)\\ =F\left(\beta_{0}+\beta_{1}Cor_{i}+\beta_{2}Hos_{i}+\ldots +\beta_{9}Nea_{i}\right)\\ =\frac{1}{1+e^{-\left(\beta_{0}+\beta_{1}Cor_{i}+\beta_{2}Hos_{i}+\ldots +\beta_{9}Nea_{i}\right)}} \end{array}$$

where *F* denotes the cumulative standard logistic distribution, β_0_ is the intercept term; β_1_-β_9_ are the parameters of the explanatory variables to be estimated.

In this paper, the willingness of the elderly to choose nursing care (Nur) was set as the dependent variable. In the questionnaire, “If there is a nursing home, would you choose to receive care there when you begin to have difficulties taking care of yourself?” was a single response question with 3 answer choices, i.e., willing, unwilling, and never considered. A value of 1 was assigned to “willing”, and 0 was assigned to the other 2 responses.

For independent variables, first, this study used the respondents’ living arrangements (Cor) to measure the intergenerational support received by the elderly, and living arrangement refers to whether the older person lives with their children. A value of 1 was assigned to the condition that the older person was living with his/her children, and 0 was assigned to the other conditions. Second, this paper used the number of hospitalizations (Hos) in the past year to measure the physical condition of the respondents. The response options for number of hospitalizations were 0, 1, 2, 3, and 4 or more, and the assigned values were 0, 1, 2, 3, and 4, respectively. Last, this paper used the question “What type of medical insurance coverage do you have?” (Ins) to assess the insurance status of the respondents; 0 indicated no insurance, and 1 indicated insurance coverage. In addition, there were other independent variables, including gender (Gen), age (Age), marital status (Mar), education level (Edu), monthly income (Min), and whether there was a nursing home near the place of residence (Nea). The variables in the model are described in Table 2.

**TABLE 2:** Variable descriptions.

Variable		Full name	Measure
Dependent variable	Nur	Nursing Care Willingness	1: Willingness; 0: No Willingness or Never thought
Explanatory variable	Cor	Co-residence	1: Coresidence; 0: Non-Coresidence
	Hos	Hospital Times	0: Zero times; 1: One time; 2: Two times; 3: Three times; 4: More than four times
	Ins	Insurance	1: Insured; 0: Uninsured
	Gen	Gender	1: Male; 0: Female
	Age	Age (years)	Retain the original data
	Mar	Marital Status	1: Married; 0: Widowed or Divorced or Never married
	Edu	Education	In the order of options 1 to 8
	Min	Monthly Income	Retain the original data
	Nea	Nursing Care Nearby	1: Yes; 0: No or unclear

## Results

This paper used a logit regression model (1) to study the effect of each factor on the willingness of the elderly to choose nursing care and determine the impact of each variable and its trend. The results are displayed in Table 3.

**TABLE 3:** Baseline regression results.

Variable	Coef.	Odds Ratio	Std. Err.	P>|z|
Cor	–0.2221	0.8008	0.1054	0.091*
Hos	–0.1113	0.8947	0.0400	0.013**
Ins	0.3418	1.4074	0.2881	0.095*
Gen	–0.3344	0.7158	0.0563	0.000***
Age	–0.0102	0.9899	0.0042	0.018**
Mar	–0.0379	0.9628	0.0901	0.685
Edu	0.2308	1.2596	0.0400	0.000***
Min	0.0001	1.0001	0.0000)	0.000***
Nea	0.2200	1.2461	0.0923	0.003***
Constant	–0.3451	0.7081	0.2761	0.376

***p<0.01, **p<0.05, *p<0.1.

The results showed that intergenerational support had a significant impact on the willingness of the elderly to choose nursing care. Compared with empty nesters or the elderly living alone, the elderly living with children were more inclined to choose to receive care at home, and their chance of choosing nursing care was 0.8 times (i.e., 20% less) that of empty nesters or the elderly living alone. Empty nesters or older people living alone have inadequate family care resources; they suffer from a long-term lack of communication and are prone to mental illness, leading to cognitive decline (van Gelder et al., 2006). Additionally, as they grow older, they may find it more challenging to live alone, or they may not completely take care of themselves. At this point, an elderly individual may choose nursing care instead of family care. Currently, the housekeeping and catering services provided by many nursing homes can significantly reduce the labor burden of the elderly, and in nursing homes, there are groups of older people with similar ages and similar experiences with whom to chat and talk, which reduces loneliness.

In terms of the physical condition of older people, compared with healthy older people, unhealthy older people tended to choose to receive care at home. However, studies have shown that older people who are unhealthy or have chronic diseases tend to choose nursing homes (Lee and Kwok, 2005). The following reasons may account for the different results. (1) The elderly in China have doubts about the level of medical services provided by nursing homes. They believe that nursing homes can only provide basic living needs and cannot provide adequate medical services. (2) Unhealthy older people need more comfort from their families, which is currently lacking in services provided by nursing homes. At present, there is not enough interaction between nursing homes and the families of the elderly; therefore, the elderly think that they have been abandoned if they live in nursing homes. (3) As the parents of only children gradually become elderly, they are more inclined to enjoy their life in nursing homes when they are in good health, thus reducing the burden to their only children.

In addition, compared with females, elderly males tended to choose to receive care at home; the likelihood of them choosing a nursing home was 0.7 times that of women. Gender had a significant impact on the willingness to receive elderly care, indicating that there is a significant gender difference in the willingness of the elderly in China to receive elderly care. Therefore, services provided through nursing care should be more diversified. There was a significant negative correlation between the willingness of the elderly to choose nursing care and age; the older the elderly were, the more likely they were to choose care at home. Older people feel lonely and insecure as they grow older and want their children to be there for them. Additionally, the elderly, who may have developed long-term routines and behaviors, do not want to leave the familiarity of their place of residence to live in a strange nursing home. The education level and the willingness of the elderly to choose elderly care were significantly correlated. The more educated an elderly individual was, the more open and inclusive he/she was, and the more receptive he/she was to nursing care. There was a significant positive correlation between the monthly income of the elderly and the willingness of the elderly to choose elderly care. The better the financial situation of the elderly, the higher their ability was to pay and the stronger their willingness was to choose nursing care. Compared with older people who did not have a nursing home near their residence, older people who did have a nursing home nearby were more likely to accept nursing elderly care. This may have occurred because older people are more willing to choose nursing homes in familiar areas. On the one hand, they are more familiar with the service level of nursing homes; on the other hand, it is convenient for family members to visit them in nursing homes. Compared with uninsured older people, insured older people had a higher probability of choosing nursing elderly care, 1.5 times (i.e., 50% higher) that of older people without any medical insurance; insurance coverage had a significant impact on the choice of mode for elderly care. Compared with uninsured older people, insured older adults with disabilities and dementia living in designated nursing homes can obtain the corresponding subsidies and professional care.

Although the probability of older people with a partner choosing a nursing home was lower than that of older people without a partner, the effect of the marital status of older people was not statistically significant, indicating that the presence or absence of a partner had no substantial impact on the willingness of the elderly to choose elderly care. This result may be related to cultural differences between China and the West. Unlike the Western family relationship with the spousal relationship as the core, in China, the parent-child relationship is the core of the family relationship. Therefore, whether an elderly individual has a partner does not cause a significant difference in their choice of elderly care (Gierveld et al., 2012).

## Robustness Checks

This paper further adopted a probit model and used a variable replacement method to conduct robustness tests; the results are shown in Tables 4, 5, respectively.

**TABLE 4:** Results for robustness test with an alternative model.

Variable	Coef.	Std. Err.	Z	P>|z|
Cor	–0.1373	0.0809	–1.70	0.089*
Hos	–0.0692	0.0277	–2.50	0.012**
Ins	0.2135	0.1253	1.70	0.088*
Gen	–0.2059	0.0490	–4.20	0.000***
Age	–0.0063	0.0027	–2.34	0.019**
Mar	–0.0222	0.0582	–0.38	0.703
Edu	0.1434	0.0196	7.32	0.000***
Min	0.0001	0.0000	3.30	0.001***
Nea	0.1377	0.0460	3.00	0.003***
Constant	–0.2172	0.2420	–0.90	0.370

***p<0.01, **p<0.05, *p<0.1.

**TABLE 5:** Results for robustness test with an alternative measure.

Variable	Coef.	Std. Err.	Z	P>|z|
Cor	–0.2395	0.1315	–1.82	0.069*
Hos	–0.1131	0.0447	–2.53	0.011**
Ins	0.3839	0.2036	1.89	0.059*
Gen	–0.3135	0.0782	–4.01	0.000***
Age	–0.0078	0.0042	–1.87	0.062*
Mar	–0.0746	0.0953	–0.78	0.434
Edu	0.2527	0.0306	8.25	0.000***
Min_new	0.00003	0.0000	3.41	0.001***
Nea	0.2181	0.0742	2.94	0.003***
Constant	–0.5374	0.3830	–1.40	0.161

***p<0.01, **p<0.05, *p<0.1.

In Table 4, the probit regression results showed that the significance of each variable did not change. In terms of the regression coefficient, non-empty-nesters and the elderly with more hospitalizations had significantly lower willingness to choose nursing homes; compared to uninsured older people, the insured elderly’s willingness to choose a nursing home was considerably higher, which is consistent with our main findings. Very old males tended to choose to receive care at home, and with increases in education level and monthly income, older people were more likely to choose nursing care. Additionally, the older people who had a nursing home nearby were more inclined to choose nursing care. The probit model results were highly consistent with the conclusions of the main tests, indicating that the regression results in this study are robust and that the research conclusions are reliable.

This paper also replaces “income from last month” (Min) with “total family income from last month” (Min_new) for the robustness test. The test results are shown in Table 5. The sign and significance of the coefficients of substitution variables and other variables did not change, which again confirms that the conclusions of this paper are reliable.

## Conclusion

With the increasing global population aging and the worsening psychological conditions of older people, physical and psychological problems involving the elderly population are constantly emerging. The elderly care mode is gradually changing from traditional family support to modern social support. Based on an empirical study of 3,513 questionnaires completed by elderly residents in 7 cities in China, this paper draws the following conclusions. (1) Intergenerational family support is a significant psychological factor influencing the willingness of the elderly to choose care. Empty nesters and older people living alone are more likely to choose nursing homes when they have difficulties taking care of themselves, indicating that China’s traditional concept of family support for elder care is still fundamental. Although the function of family support for the care of the elderly is weakening, the spiritual consolation provided by family members is of great importance to the elderly. Older people are more willing to receive care in a familiar living environment. (2) From the perspective of the physical health of older people, older people with more hospital stays tend to choose to receive care at home. It can be inferred that the healthier older people are present, the higher the tendency is of choosing nursing care when they begin to have difficulties taking care of themselves. Nursing homes are responsible for the specialized long-term care of disabled and semi-disabled elderly. However, at present, older people do not trust the humanized and professional services provided by nursing homes, leading to a mismatch between supply and demand. Through empirical analysis, this paper concludes that older people with weak intergenerational support and good health are more willing to choose nursing homes when they begin to have difficulty taking care of themselves. In addition, elderly females who are relatively young, have a high level of education, have a high income, have a nursing home near their residence, and are already covered by medical insurance are more willing to choose nursing care.

The main contribution of this paper is to use authoritative data obtained through scientific sampling to analyze the factors that influence the willingness of the elderly in China to choose nursing care. Based on the theory of healthy aging and conclusions from this study, the following recommendations are provided. First, the concept of people-oriented care should be established, i.e., provide a friendly living environment for the elderly, feed the elderly with multilevel care services, including physical, psychological, and social interactions, under the premise of maintaining self-esteem, self-reliance, and independence, and protect the legitimate rights, interests, and fundamental rights of the elderly.

Second, the long-term care insurance system needs to be further improved to provide financial security for the elderly with disabilities and dementia. Currently, the number of beds in nursing homes in China continues to increase, while the occupancy rate is not high; the main reason for this discrepancy is that the income earned by elderly individuals is not enough to cover the costs of a nursing home. Most older people do not choose nursing homes, which is not entirely a matter of their preference but more of their inability to pay the cost. This also causes an operational dilemma for nursing homes, especially private nursing homes (Tang and Feng, 2015). The long-term care insurance system can assume certain economic risks for the elderly when they have difficulties in taking care of themselves, can help to transform the potential demand of nursing care for the elderly into effective demand, and can promote the sustainable development of nursing homes.

Third, nursing homes should integrate teams of interdisciplinary professionals. Elderly care involves sociology, medicine, rehabilitation, psychology, social work, and nursing. In particular, the elderly with disabilities and dementia, who have multiple comorbidities and cannot take care of themselves, require psychological comfort and social support. The multilevel needs of the elderly interact with each other, and different older people have commonalities and heterogeneity. The idea of integration is used to provide all-round, diverse, and high-quality services. The critical role of nursing homes in the long-term care service system should be fully realized to extend professional services to communities and families.

Fourth, the medical services in nursing homes should be actively promoted. In its interpretation of healthy aging, the WHO emphasizes the importance of the functional ability of the elderly. This study finds that unhealthy older people do not trust the service quality of nursing homes, affecting the willingness of those older people to choose nursing homes. Most older people in nursing homes have multiple comorbidities; therefore, authoritative medical treatment, proper treatment of sudden illness, and scientifically based rehabilitation of physical functions are essential factors for the elderly to choose nursing homes.

Fifth, nursing homes should pay attention to humanistic care and family support. A nursing home is a place where older people live, and older people need emotional care and need to express themselves. This study finds that the more physically unhealthy the elderly are, the less likely they are to leave home because older people need a sense of security and belonging provided by their families after their physical function deteriorates. Therefore, nursing homes should be conveniently located within communities so that family members can visit elderly family members. The environmental design of nursing homes must create the sense of a warm and safe home, a design that requires caregivers to have patience and provide love to make all elderly residents feel cared for and valued, even when they are away from home. Nursing homes should also establish close contact with the family members of elderly residents so that the family members can understand and actively participate in the life of their elderly family members. Family affection plays a vital role in maintaining different psychological functions in elderly individuals.

## Data Availability Statement

The data analyzed in this study is subject to the following licenses/restrictions: The data used to support the findings of this study is available upon request. Requests to access these datasets should be directed to CW, cheng2676@163.com.

## Author Contributions

CW, FZ, CP, SG, XG, and DY contributed to conception and design of the study. CW, FZ, and XG organized the database and performed the statistical analysis. All authors wrote the first draft of the manuscript, contributed to manuscript revision, read, and approved the submitted version.

## Conflict of Interest

The authors declare that the research was conducted in the absence of any commercial or financial relationships that could be construed as a potential conflict of interest.

## Publisher’s Note

All claims expressed in this article are solely those of the authors and do not necessarily represent those of their affiliated organizations, or those of the publisher, the editors and the reviewers. Any product that may be evaluated in this article, or claim that may be made by its manufacturer, is not guaranteed or endorsed by the publisher.
